# Supplementary figures and images for: mGluR7 allosteric modulator AMN082 corrects protein synthesis and pathological phenotypes in FXS
Source: EMBO Mol Med. 2024 Feb 19;16(3):5. doi: 10.1038/s44321-024-00038-w (PMC10940663; doi:10.1038/s44321-024-00038-w)

Figure: 1B

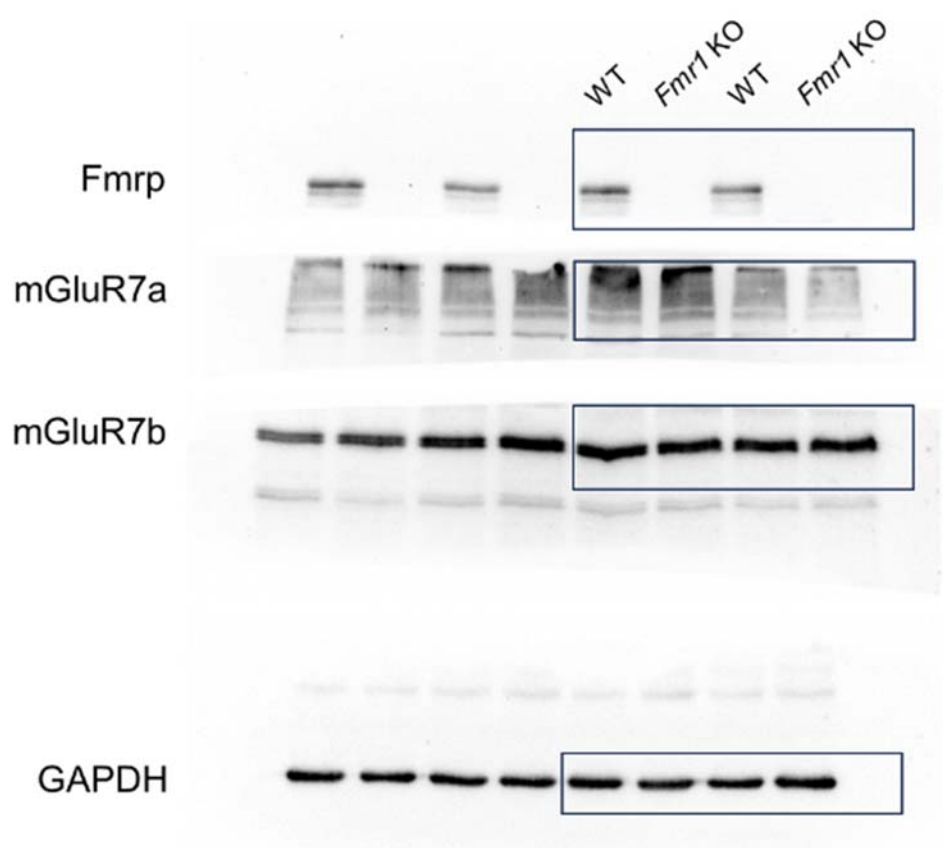

Supplement: Supplementary file 1 — Source Data Fig. 1 [file 44321_2024_38_MOESM1_ESM.zip › Figure 1 Source Data/1B/Western 1B.pdf]

Figure: 1C

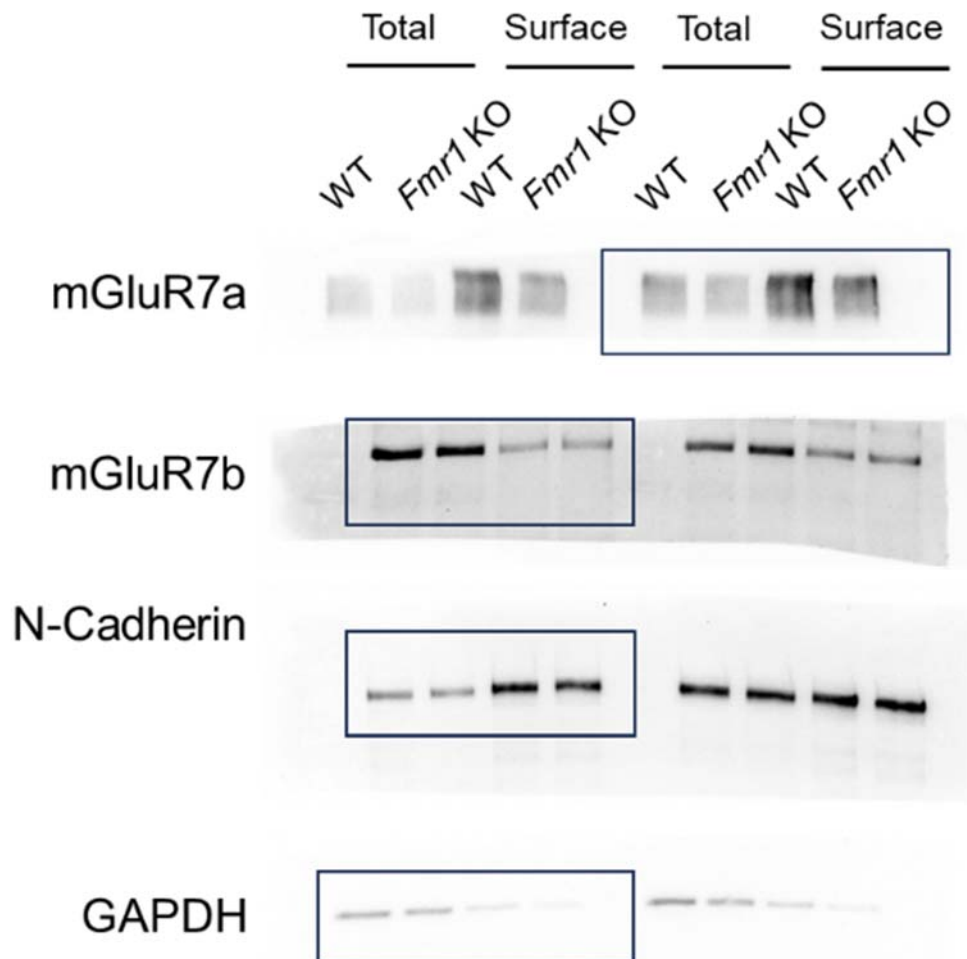

Supplement: Supplementary file 1 — Source Data Fig. 1 [file 44321_2024_38_MOESM1_ESM.zip › Figure 1 Source Data/1C/Western 1C.pdf]

Figure: 1D

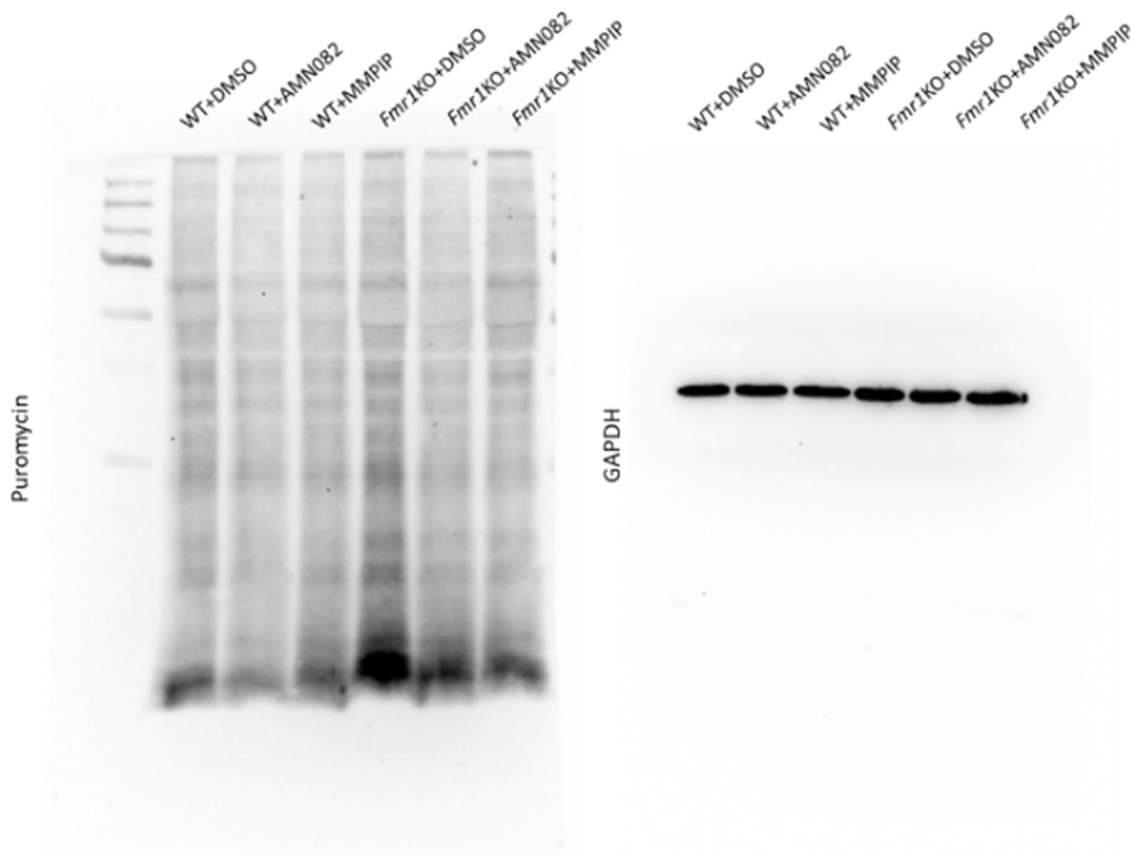

Supplement: Supplementary file 1 — Source Data Fig. 1 [file 44321_2024_38_MOESM1_ESM.zip › Figure 1 Source Data/1D/Western 1D.pdf]

Figure: 1E

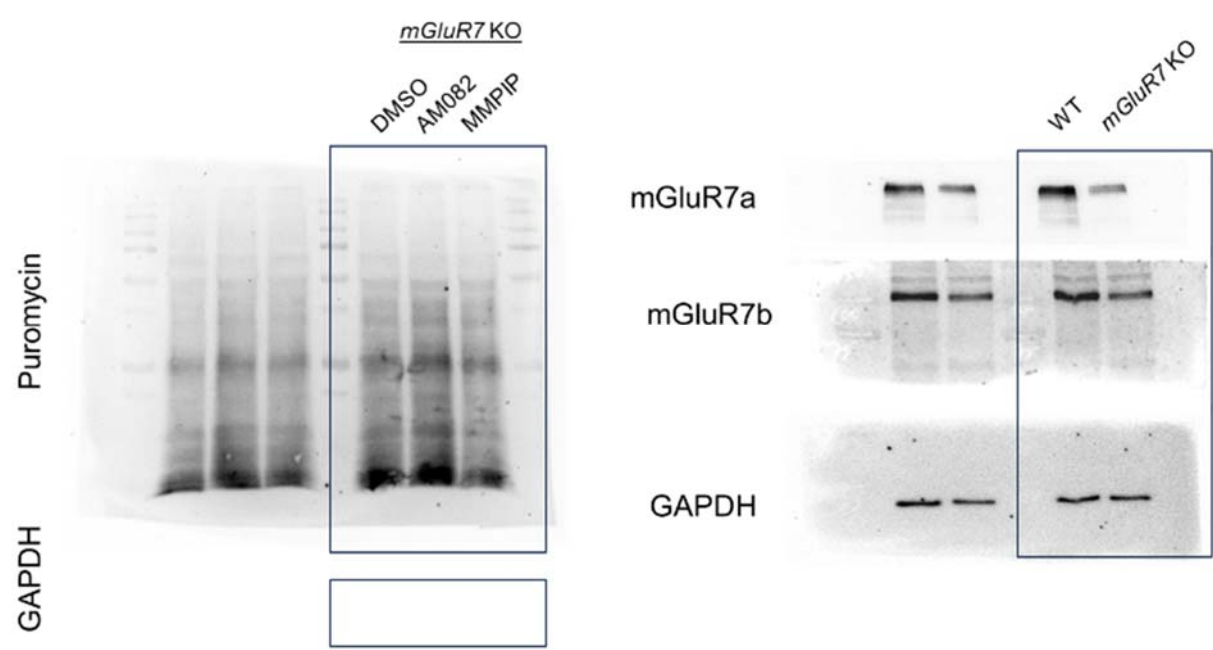

Supplement: Supplementary file 1 — Source Data Fig. 1 [file 44321_2024_38_MOESM1_ESM.zip › Figure 1 Source Data/1E/Western 1E.pdf]

Figure: 1F

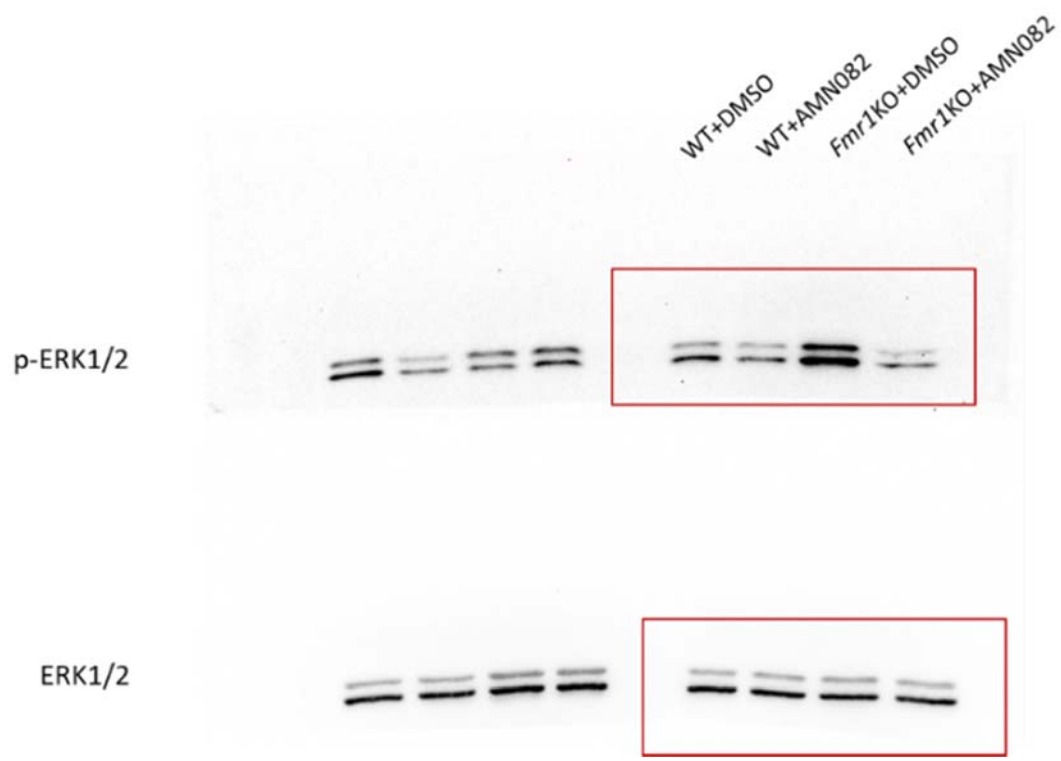

Supplement: Supplementary file 1 — Source Data Fig. 1 [file 44321_2024_38_MOESM1_ESM.zip › Figure 1 Source Data/1F/Western 1F.pdf]

Figure: 1G

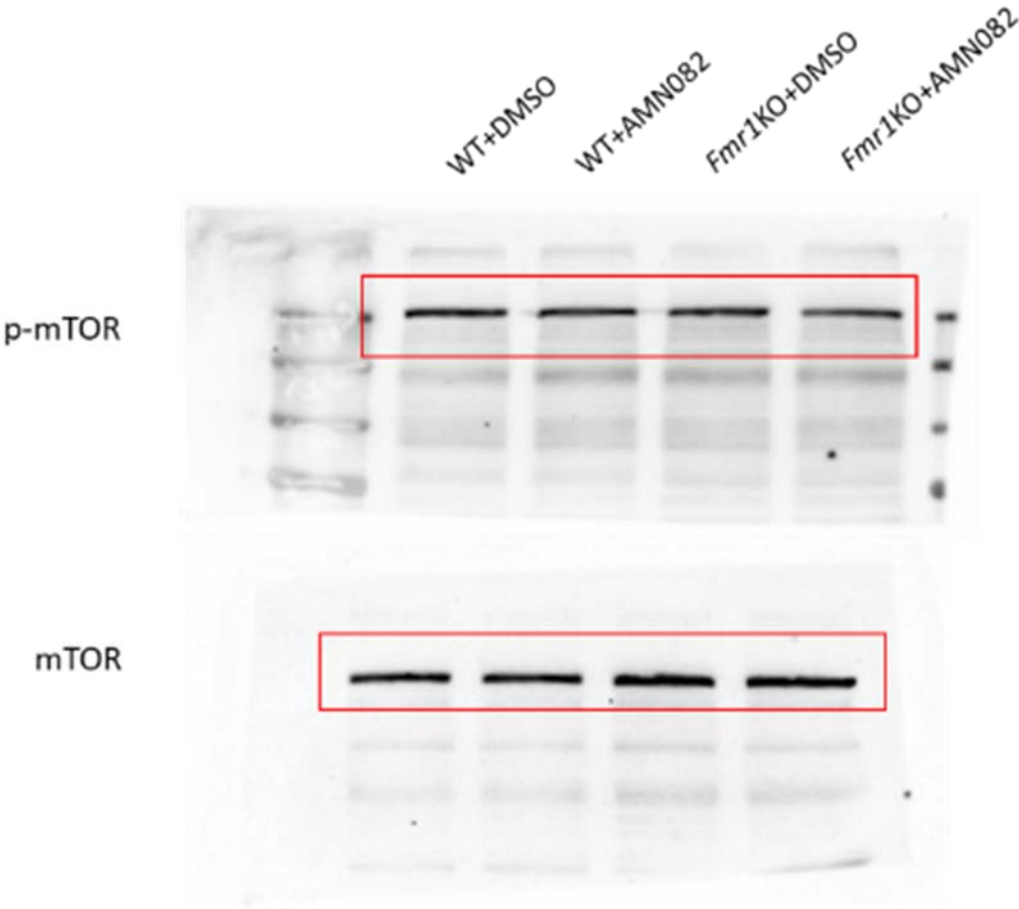

Supplement: Supplementary file 1 — Source Data Fig. 1 [file 44321_2024_38_MOESM1_ESM.zip › Figure 1 Source Data/1G/Western 1G.pdf]

Figure: 1H

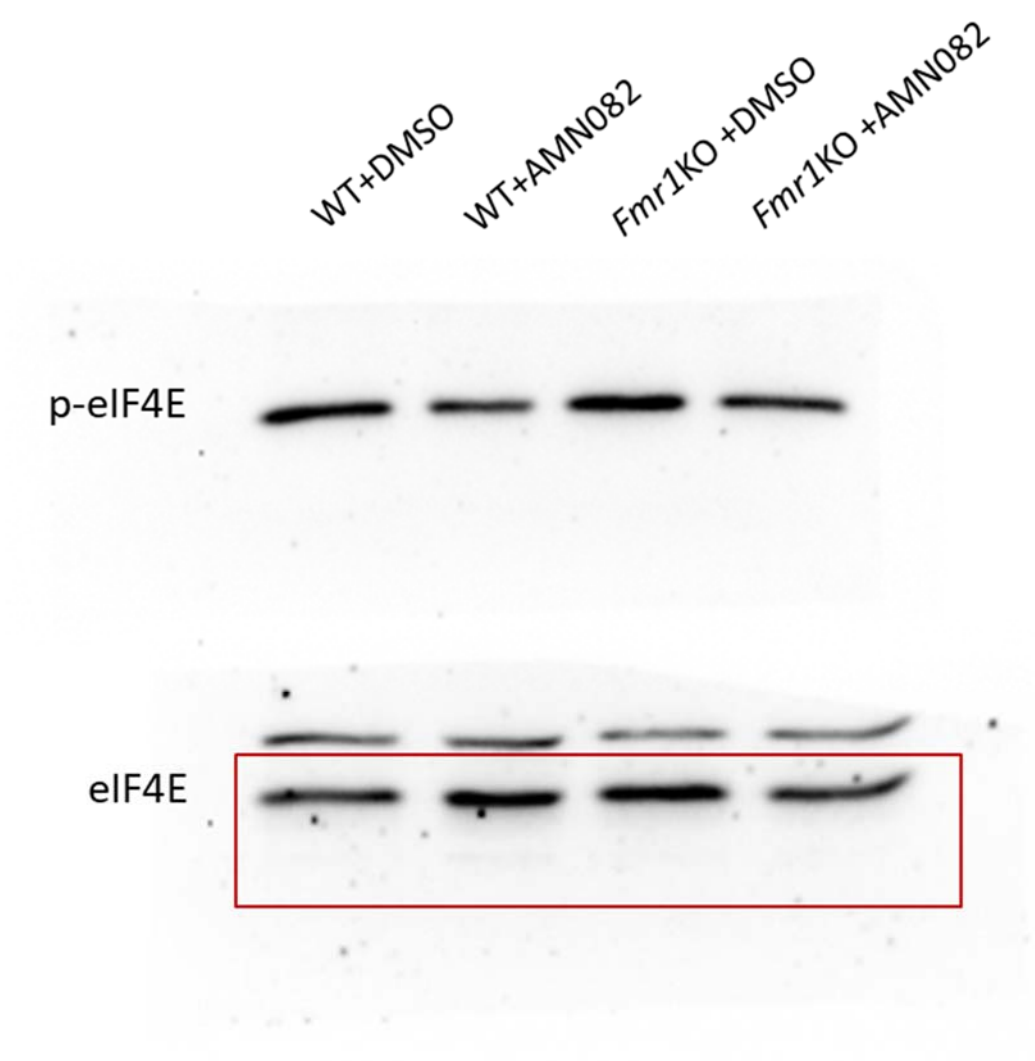

Supplement: Supplementary file 1 — Source Data Fig. 1 [file 44321_2024_38_MOESM1_ESM.zip › Figure 1 Source Data/1H/Western 1H.pdf]

Figure: 1I

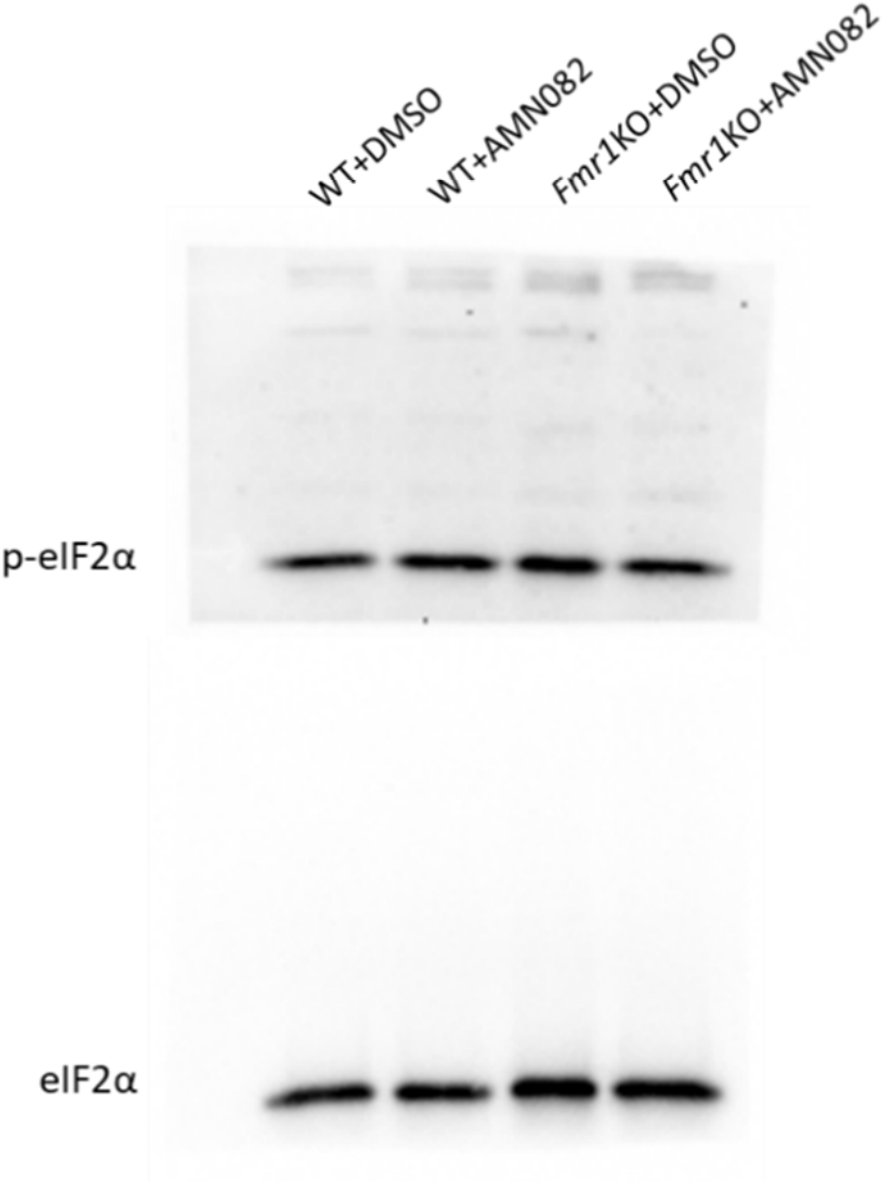

Supplement: Supplementary file 1 — Source Data Fig. 1 [file 44321_2024_38_MOESM1_ESM.zip › Figure 1 Source Data/1I/Wesern 1I.pdf]

Figure: 1J

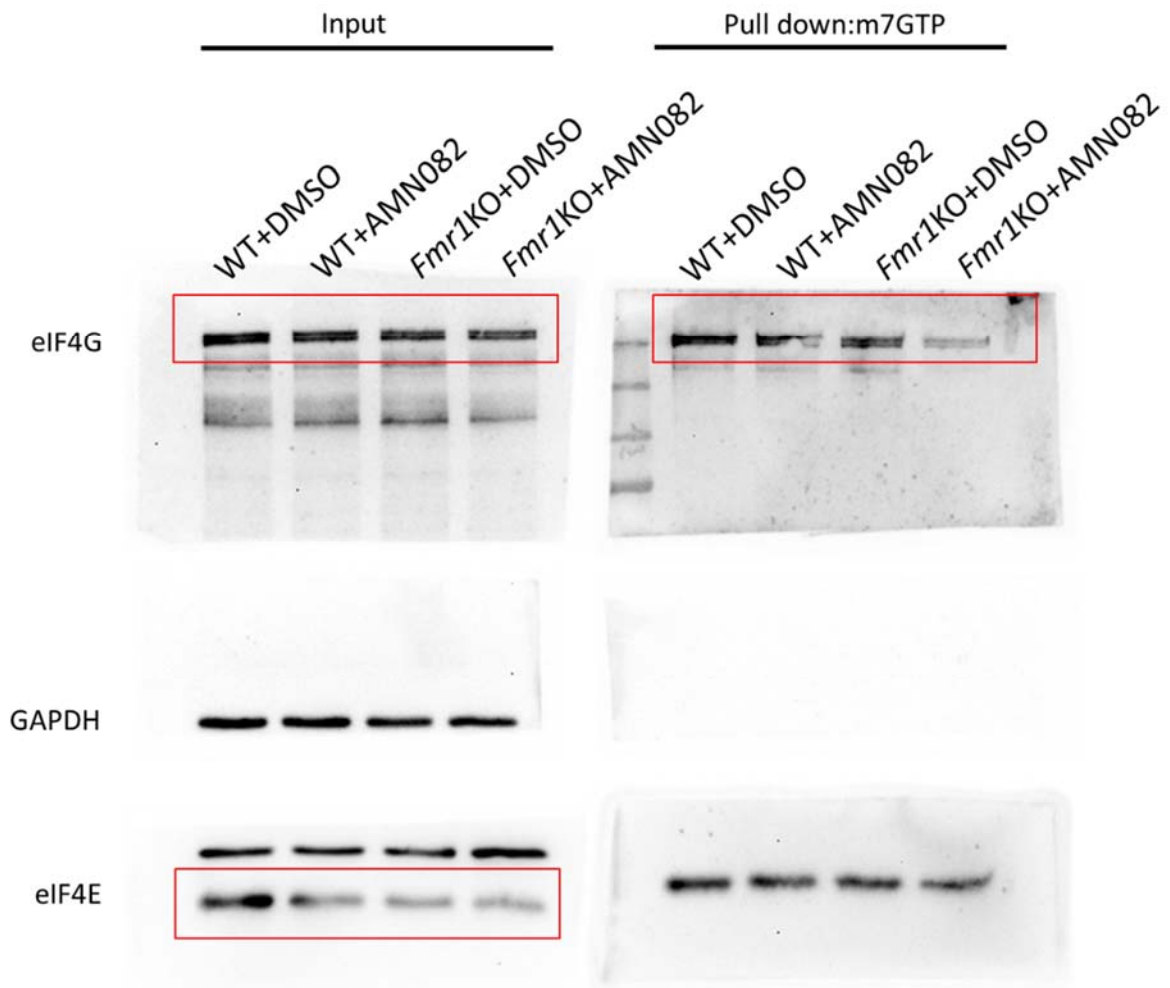

Supplement: Supplementary file 1 — Source Data Fig. 1 [file 44321_2024_38_MOESM1_ESM.zip › Figure 1 Source Data/1J/Western 1J.pdf]

Figure: 2 A

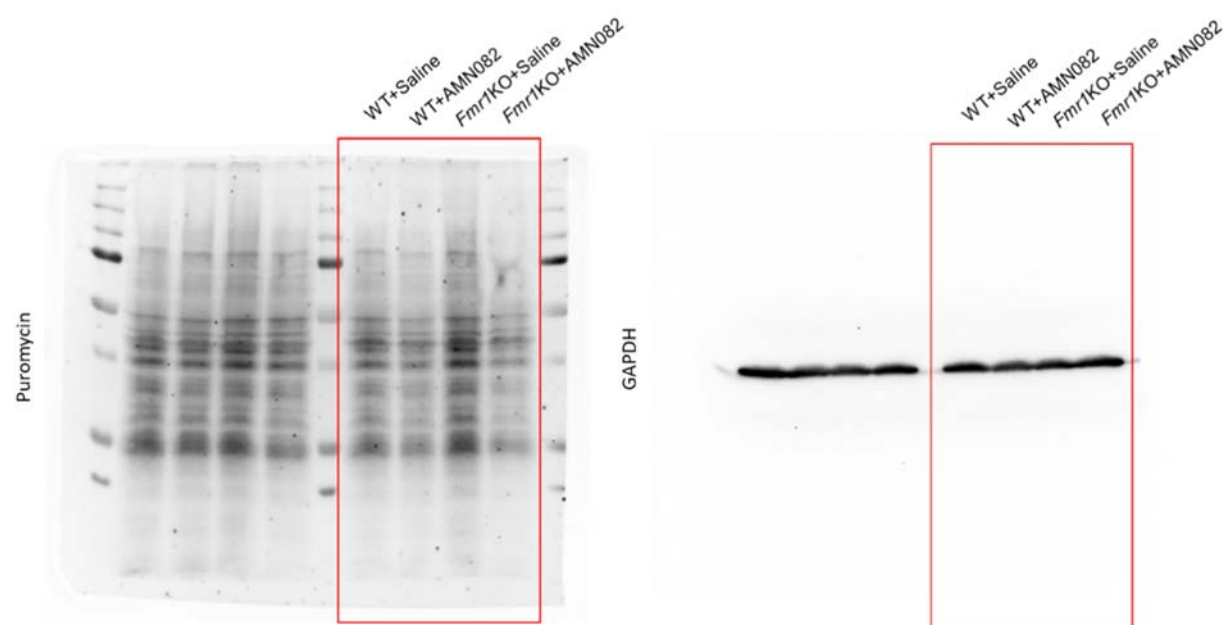

Supplement: Supplementary file 2 — Source Data Fig. 2 [file 44321_2024_38_MOESM2_ESM.zip › Figure 2 Source Data/2A/Western 2A.pdf]

Figure: 2B

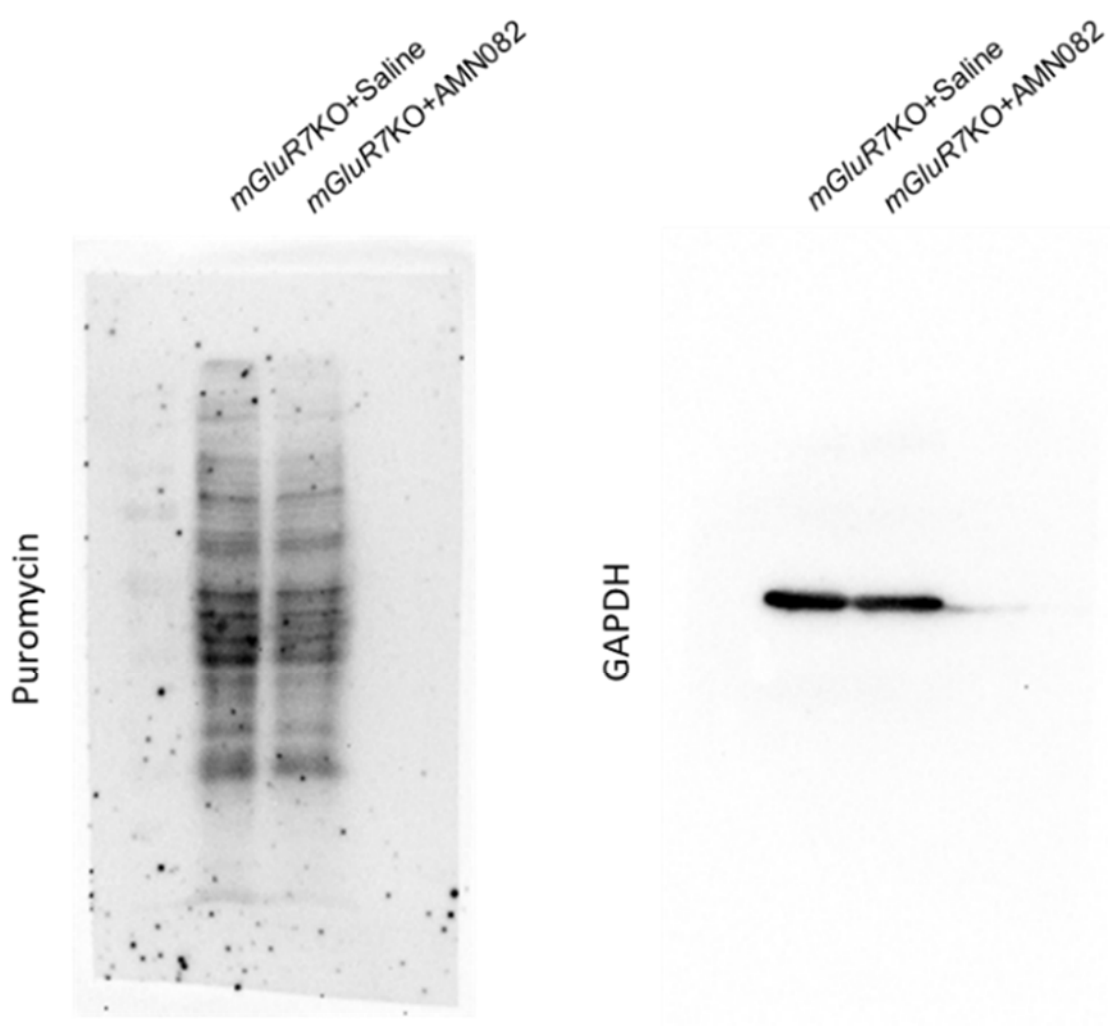

Supplement: Supplementary file 2 — Source Data Fig. 2 [file 44321_2024_38_MOESM2_ESM.zip › Figure 2 Source Data/2B/Western 2B.pdf]

Figure: 2C

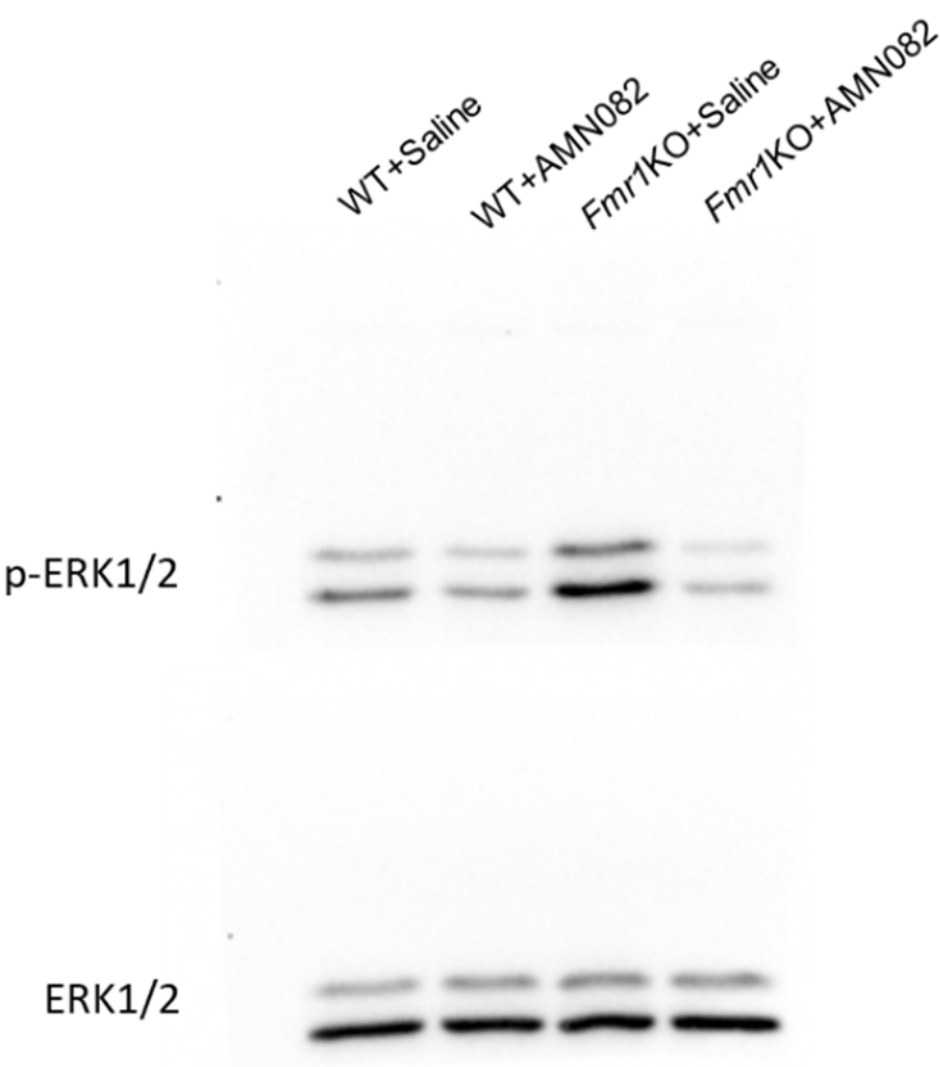

Supplement: Supplementary file 2 — Source Data Fig. 2 [file 44321_2024_38_MOESM2_ESM.zip › Figure 2 Source Data/2C/Western 2C.pdf]

Figure: 2D

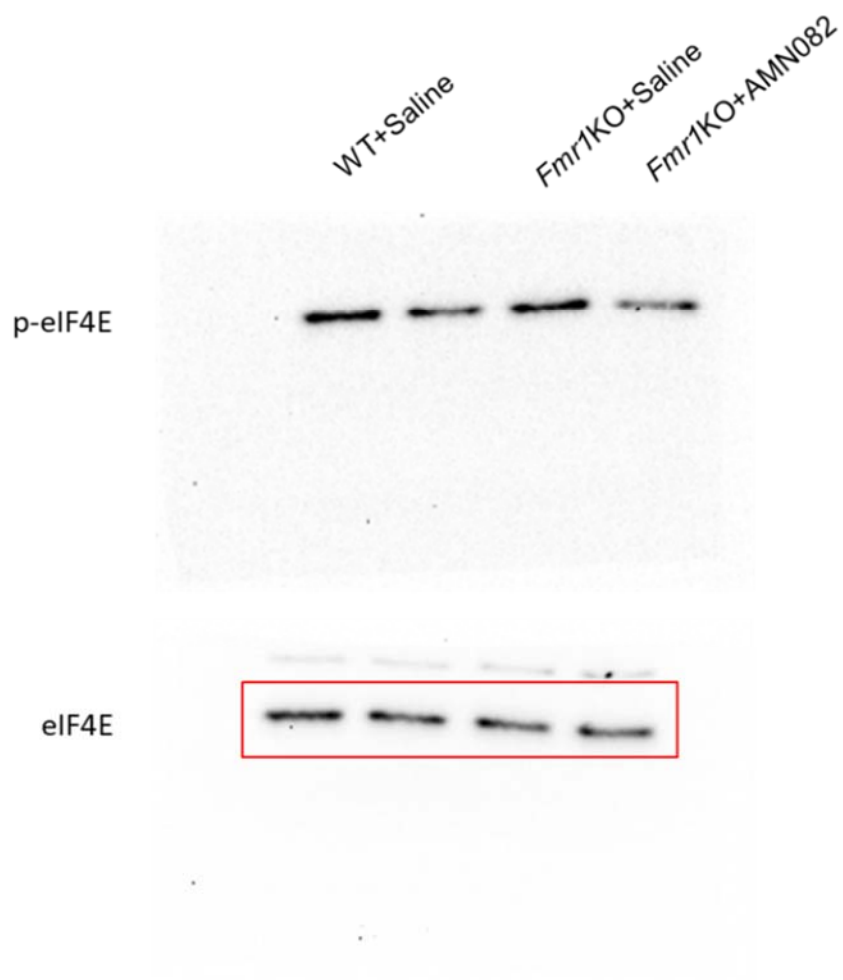

Supplement: Supplementary file 2 — Source Data Fig. 2 [file 44321_2024_38_MOESM2_ESM.zip › Figure 2 Source Data/2D/Western 2D.pdf]

Figure: EV1

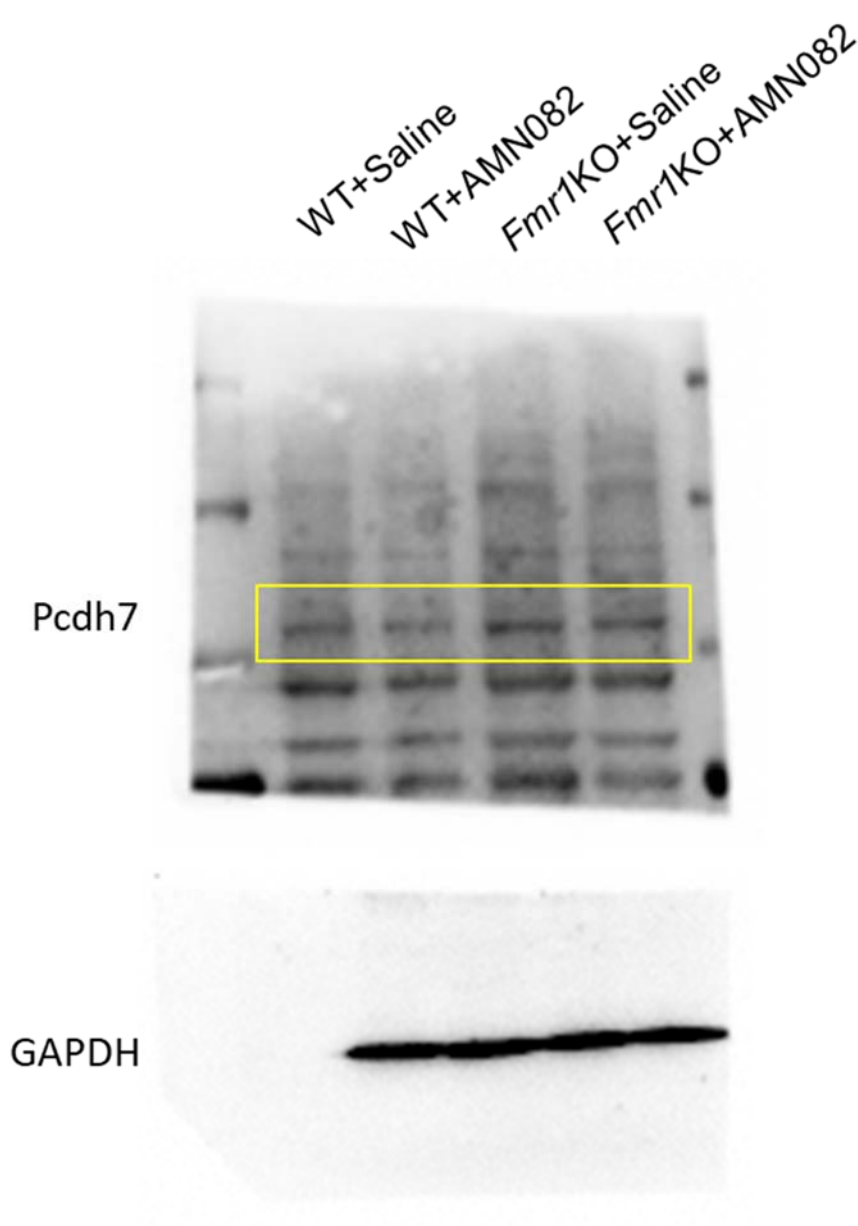

Supplement: Supplementary file 6 — Source Data Fig. EV1 [file 44321_2024_38_MOESM6_ESM.zip › Figure EV1 Source Data/Western EV1.pdf]
