# Supplementary material for: mGluR7 allosteric modulator AMN082 corrects protein synthesis and pathological phenotypes in FXS
Source: EMBO Mol Med. 2024 Feb 19;16(3):5. doi: 10.1038/s44321-024-00038-w (PMC10940663; doi:10.1038/s44321-024-00038-w)
Supplement: Supplementary file 8 — Expanded View Figures [file 44321_2024_38_MOESM8_ESM.pdf]

Expanded View Figure

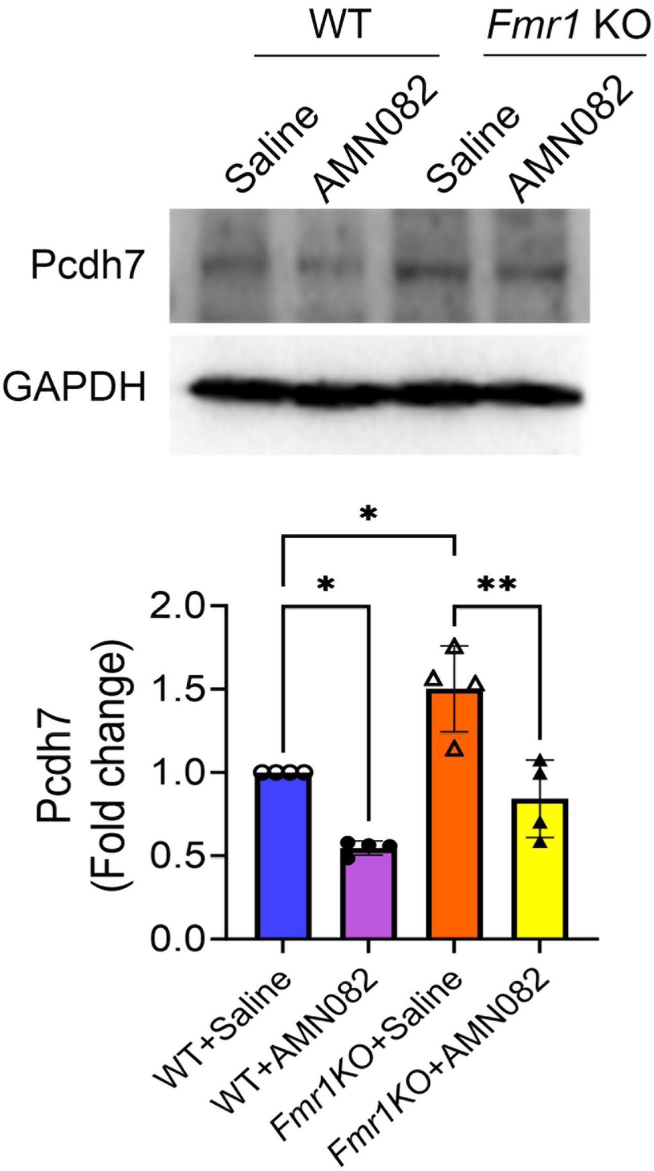

Figure EV1. Activation of mGluR reduced the levels of an Fmrp target protein Pcdh7 in the WT and *Fmr1* KO hippocampus.

Top: Representative blot showing expression of Pcdh7 in the hippocampal lysates of 6–8-week-old WT and *Fmr1* KO mice injected with saline or AMN082 (1 mg/kg). Bottom: Plot showing the quantification of band intensities of Pcdh7 from 4 independent sets of experiments expressed as fold change. Data were analyzed using Two-way ANOVA with Tukey's test and presented as mean +/- SEM. WT+Saline vs WT + AMN082, $p = 0.0213$; WT+Saline vs *Fmr1*KO +Saline, $p = 0.0120$; *Fmr1*KO+Saline vs *Fmr1*KO + AMN082, $p = 0.0021$. Source data are available online for this figure.

 © The Author(s)
